# Healthcare Utilization Differences Among Primary Care Patients Using Telemedicine in the Veterans Health Administration: a Retrospective Cohort Study

**DOI:** 10.1007/s11606-023-08472-1

**Published:** 2024-01-22

**Authors:** Amy M. J. O’Shea, Kailey Mulligan, Paige Carlson, Bjarni Haraldsson, Matthew R. Augustine, Peter J. Kaboli, Stephanie L. Shimada

**Affiliations:** 1 VA Office of Rural Health, Veterans Rural Health Resource Center-Iowa City (VRHRC-IC), Iowa City VA Healthcare System, Iowa City, IA 52246-2208 USA; 2 Center for Access and Delivery Research and Evaluation (CADRE), Iowa City VA Healthcare System, Iowa City, IA 52246-2208 USA; 3 Department of Internal Medicine, University of Iowa Carver College of Medicine, Iowa City, IA 52242 USA; 4 Department of Biostatistics, University of Iowa College of Public Health, Iowa City, IA 52241 USA; 5 Geriatric Research Education and Clinical Center, James J Peters VA Medical Center, Bronx, NY USA; 6 Department of Medicine, The Icahn School of Medicine at Mount Sinai, New York, NY USA; 7 Center for Healthcare Organization and Implementation Research (CHOIR) at the Bedford VA Medical Center, Bedford, MA USA; 8 Department of Health Law, Policy and Management, Boston University School of Public Health, Boston, MA USA; 9 Division of Health Informatics and Implementation Science, Department of Population and Quantitative Health Sciences, University of Massachusetts Chan Medical School, Worcester, MA USA

**Keywords:** telemedicine, COVID-19, internet access, primary care, veterans

## Abstract

**Background:**

The COVID-19 pandemic encouraged telemedicine expansion. Research regarding follow-up healthcare utilization and primary care (PC) telemedicine is lacking.

**Objective:**

To evaluate whether healthcare utilization differed across PC populations using telemedicine.

**Design:**

Retrospective observational cohort study using administrative data from veterans with minimally one PC visit before the COVID-19 pandemic (March 1, 2019–February 28, 2020) and after in-person restrictions were lifted (October 1, 2020–September 30, 2021).

**Participants:**

All veterans receiving VHA PC services during study period.

**Main Measures:**

Veterans’ exposure to telemedicine was categorized as (1) in-person only, (2) telephone telemedicine (≥ 1 telephone visit with or without in-person visits), or (3) video telemedicine (≥ 1 video visit with or without telephone and/or in-person visits). Healthcare utilization 7 days after index PC visit were compared. Generalized estimating equations estimated odds ratios for telephone or video telemedicine versus in-person only use adjusted for patient characteristics (e.g., age, gender, race, residential rurality, ethnicity), area deprivation index, comorbidity risk, and intermediate PC visits within the follow-up window.

**Key Results:**

Over the 2-year study, 3.4 million veterans had 12.9 million PC visits, where 1.7 million (50.7%), 1.0 million (30.3%), and 649,936 (19.0%) veterans were categorized as in-person only, telephone telemedicine, or video telemedicine. Compared to in-person only users, video telemedicine users experienced higher rates per 1000 patients of emergent care (15.1 vs 11.2; *p* < 0.001) and inpatient admissions (4.2 vs 3.3; *p* < 0.001). In adjusted analyses, video versus in-person only users experienced greater odds of emergent care (OR [95% CI]:1.18 [1.16, 1.19]) inpatient (OR [95% CI]: 1.29 [1.25, 1.32]), and ambulatory care sensitive condition admission (OR [95% CI]: 1.30 [1.27, 1.34]).

**Conclusions:**

Telemedicine potentially in combination with in-person care was associated with higher follow-up healthcare utilization rates compared to in-person only PC. Factors contributing to utilization differences between groups need further evaluation.

**Supplementary Information:**

The online version contains supplementary material available at 10.1007/s11606-023-08472-1.

## INTRODUCTION

Telemedicine expanded rapidly during the COVID-19 pandemic to ensure patients received care in the safest possible setting.^1, 2^ Within the Veterans Health Administration (VHA), primary care (PC) telemedicine is delivered by telephone or video. While patient-level barriers exist to adopt telemedicine including older age, lower income and education, as well as rural residence,^3, 4^ the VHA has provided qualified veterans with Internet-enabled devices and negotiated with telecommunication companies to provide free unlimited data for VHA video visits.^5–7^ As the pandemic has receded, telemedicine continues to play a role in patient care.

VHA was an early telemedicine adopter with programs focused on increasing access to primary and subspecialty care.^9–13^ As pandemic restrictions continued, VHA telemedicine surged.^2, 8^ In specific populations, such as patients with type 2 diabetes, heart failure, or COPD, there is evidence that home telehealth programs, using remote monitoring tools (blood pressure cuffs, pulse oximeters, etc.), can lead to decreased hospitalizations and ED visits.^14–16^ However, there is a lack of research regarding follow-up healthcare utilization following PC telemedicine, including subsequent visits to PC, emergent care (either urgent care or the emergency department), and inpatient and ambulatory care sensitive condition (ACSC) admissions. Prior studies yielded conflicting evidence with some supporting that telemedicine initiation leads to more follow-up appointments^17^ while others showed no less utilization and fewer hospitalizations.^18, 19^ Though patients and providers are generally satisfied telemedicine,^20, 21^ if such care increases downstream healthcare utilization, patients may be burdened by increased costs and care delays.

The study objective was to evaluate whether follow-up healthcare utilization differed across PC populations using telemedicine (i.e., video and telephone) compared to in-person. We hypothesized patients would increase PC telemedicine use after pandemic onset, and those using telemedicine would have higher overall PC visit rates. We additionally hypothesized that subsequent healthcare system use (i.e., emergent care, inpatient, or ACSC admissions) within 7 days of an index PC visit would be comparable to those using only in-person PC visits. This research can provide insights into how delivery method may impact downstream healthcare.

## METHODS

### Study Design

This is a retrospective observational cohort study of PC VHA outpatient visits before the COVID-19 pandemic (March 1, 2019–February 28, 2020) and after the re-opening of VHA medical centers to in-person visits (October 1, 2020–September 30, 2021). Outpatient visits between March 1, 2020, through September 30, 2020, were excluded, because in-person restrictions dramatically decreased overall healthcare utilization and necessitated telemedicine use. VHA facilities lifted restrictions at varying times during the pandemic. This study followed the Strengthening the Reporting of Observational Studies in Epidemiology (STROBE) reporting guideline^22^and was approved by the University of Iowa Institutional Review Board and the Iowa City VA Healthcare System Research and Development Committee. It was conducted without direct patient contact using data routinely collected in the electronic health record. It was deemed of minimal risk; therefore, a waiver of informed consent was obtained.

### Data Sources

Data were managed in the Veterans Informatics and Computing Infrastructure, a secure integrated system which includes all VHA administrative data and electronic health records. Patient-level data, including demographics, date, and delivery method for PC visits, as well as visits to the emergency department or urgent care, was obtained from the Corporate Data Warehouse (CDW) outpatient domain. The date of inpatient admissions was similarly identified using the CDW inpatient domain. The 2010 Census Bureau TIGER/Line shapefile contains geographic entity codes, including census block, census block group, and census tract. These data were spatially merged with the fiscal year-specific latitude and longitude of each veteran’s home address to identify their census block-based broadband availability using the December 2019 Federal Communications Commission (FCC) Fixed Broadband data,^23^ their census block group-based area deprivation index (ADI),^24^ a ranking of neighborhood socioeconomic disadvantage, and their census tract-based social vulnerability index (SVI),^25^ a Centers for Disease Control and Prevention measure of a community’s ability to respond to a hazardous event. Residential rurality classification was obtained from the Planning Systems Support Group (PSSG).

### Patient Population

We established a cohort of veterans who used outpatient VHA PC prior to the pandemic (March 1, 2019–February 28, 2020) and after the re-opening of VHA medical centers (October 1, 2020–September 30, 2021). This ensured the comparison of healthcare utilization among the same group of veterans. PC encounters were categorized using stop codes, a pair of proprietary three-digit codes assigned to each outpatient encounter (Appendix 1). To be included, a veteran was required to have at least one PC visit, regardless of visit modality (i.e., in-person, telephone, or video) in each study period. We excluded care received at residential rehabilitation centers, nursing homes, or domiciliary.

### Telemedicine Use

Our primary exposure was PC telemedicine (i.e., telephone or video) use. An index visit was the first PC visit within the study period, with each subsequent index visit occurring at least 7 days later. Index visits were not restricted by visit modality. Within the 7-day follow-up period, we assessed the number of days on which a PC visit occurred, categorized by visit modality (e.g., in-person, telephone, or video). Veterans were categorized into three mutually exclusive modality groups using index and intermediate PC visits throughout the study period overall as (1) in-person only, (2) telephone telemedicine (≥ 1 telephone visit with or without in-person visits), or (3) video telemedicine (≥ 1 video visit with or without telephone and/or in-person visits).

### Outcomes

We studied three outcomes in the 7 days following an index PC visit: (1) emergent care (i.e., emergency department or urgent care visits), (2) any inpatient admission, and (3) any ACSC admission. Conditions included as ACSCs were community-acquired pneumonia, urinary tract infections, long- and short-term diabetes complications, lower-extremity amputation among diabetic patients, chronic obstructive pulmonary disease or asthma in older adults, heart failure, hypertension, and admission for asthma among young adults with diabetes.

### Covariates

Patient demographics included age, sex, race, ethnicity, broadband availability, ADI, and SVI. Residential rurality was identified using the geocoded location of the patient’s home via Rural Urban Commuting Area codes and dichotomized into urban and rural (i.e., rural, highly rural, and insular categories).^26^ Race and ethnicity were self-reported. Race was categorized as American Indian/Alaska Native, Asian, Black/African American, Native Hawaiian/Pacific Islander, White, or Missing. Ethnicity was reported as being Hispanic, Not Hispanic, or Missing. Broadband availability was categorized according to download and upload speeds as inadequate (download ≤ 25 Mbps; upload ≤ 3 Mbps), adequate (download ≥ 25 Mbps and < 100 Mbps; upload ≥ 5 Mbps and < 100 Mbps), or optimal (download and upload ≥ 100Mbps). A minimum of 25 Mbps download and 3 Mbps upload are recommended for video telemedicine. We also calculated a comorbidity score based on the previous year using the methodology described by Quan et al. (2011), with a modification to allow for two outpatient diagnoses, as well as a single inpatient diagnosis.

### Statistical Analyses

Demographic and healthcare utilization rates were compared to the in-person modality group (i.e., referent category) using chi-square or *t*-tests. Generalized estimating equations evaluated the difference in hospital utilization and PC visit modality group. The dependent binary variable indicated ever use of emergent care visit, any inpatient admission, or any ACSCs admissions, respectively, within 7 days of index PC visit. Independent variables included a binary indicator for study period (i.e., pre-pandemic vs. after in-person restrictions were lifted), a categorical variable for visit modality group, and their interaction. Odds ratios and 95% confidence intervals are reported for a model with and without the interaction term. Both models adjusted for patient characteristics and the number of intermediate PC visits within the 7-day follow-up window. All logistic regressions used the binomial model structure with a logit link function, an independent error structure, and standard errors clustered at the veteran level. All hypothesis tests were two-sided with an a priori 0.05 level of significance.

### Sensitivity Analyses

Similar analyses considered a variety of follow-up windows from index visit (e.g., 3, 14, and 28 days), as well as the exclusion of any PC visit with a COVID-19 diagnosis.

The authors had full access to and take full responsibility for the integrity of the data. All analyses were conducted using SAS® statistical software version 9.2 (Cary, NC) and SQL Server Management, version 18.8.

## RESULTS

During the study, 3,420,034 unique veterans obtained PC at 855 PC clinics of whom 90.7% were male, 73.1% White, and 64.2% urban-residing, with a mean age of 61.7 (SD = 15.3) years (Table 1). Among the study cohort, 50.7% used only in-person care, 30.3% experienced ≥ 1 telephone visit, and 19.0% used ≥ 1 video visit. Veterans using some video versus only in-person care were younger (mean [SD], 56.5 [15.6] vs. 63.1 [15.0] years; *p* < 0.001), and more likely to be Black (23.7% vs. 16.0%; *p* < 0.001), female (15.0% vs. 8.1%; *p* < 0.001), and urban-residing (76.0% vs. 61.0%; *p* < 0.001) with more optimal broadband availability (40.8% vs. 32.2%; *p* < 0.001). The telephone telemedicine and in-person only groups were demographically similar, despite small, statistically significant differences.

**Table 1:** Unadjusted Comparisons of Participant Demographics among Telephone or Video Telemedicine Modality Groups, Respectively, Compared to the In-person Only Modality Group

	In-person only*	Telephone telemedicine	Video telemedicine
Sample size, *N* (%)	1,733,611 (50.7)	1,036,487 (30.3)	649,936 (19.0)
Gender, *N* (%)			
Female	139,966 (8.1)	81,012 (7.8)	97,849 (15.0)
Race, *N* (%)			
White	1,305,609 (75.3)	761,123 (73.4)	431,013 (66.3)
Black or African American	277,359 (16.0)	178,076 (17.2)	153,812 (23.7)
Asian	15,674 (0.9)	11,154 (1.1)	10,819 (1.7)
Native Hawaiian or Pacific Islander	14,783 (0.9)	11,698 (1.1)	7,430 (1.1)
American Indian/Alaska Native	13,977 (0.8)	10,277 (1.0)	5,231 (0.8)
Missing	106,209 (6.1)	64,159 (6.2)	41,631 (6.4)
Ethnicity, *N* (%)			
Not Hispanic or Latino	1,571,885 (90.7)	931,554 (89.9)	565,460 (87.0)
Hispanic or Latino	100,148 (5.8)	67,346 (6.5)	61,198 (9.4)
Missing	61,578 (3.6)	37,587 (3.6)	23,278 (3.6)
Rurality^†^, *N* (%)			
Rural	676,725 (39.0)	391,080 (37.7)	155,892 (24.0)
Broadband category, *N* (%)			
Optimal	557,154 (32.2)	336,586 (32.6)	264,502 (40.8)
Adequate	990,264 (57.2)	600,348 (58.1)	347,923 (53.6)
Inadequate	184,209 (10.6)	96,913 (9.4)	36,639 (5.6)
Comorbidity score, mean (SD)	0.4 (0.9)	0.5 (1.0)	0.4 (1.0)
Age, mean (SD)	63.1 (15.0)	62.5 (14.8)	56.5 (15.6)
ADI^‡^, mean (SD)	56.3 (24.7)	54.8 (24.5)	50.2 (25.7)
SVI^§^: overall tract rank, mean (SD)	0.3 (12.3)	0.3 (13.4)	0.2 (16.0)

^*^All comparisons versus the in-person only group were significant at the < 0.001 level

^†^Includes patients who lived in a rural area at any time during the study period

^‡^Area deprivation index, ranks neighborhoods by socioeconomic disadvantage on a scale of 0–100 with lower rankings indicating less social disadvantage

^§^Social vulnerability index, the overall census tract ranking determined by the U.S. Centers for Disease Control and Prevention to identify communities at higher social risk following a disaster

**Figure 1:**
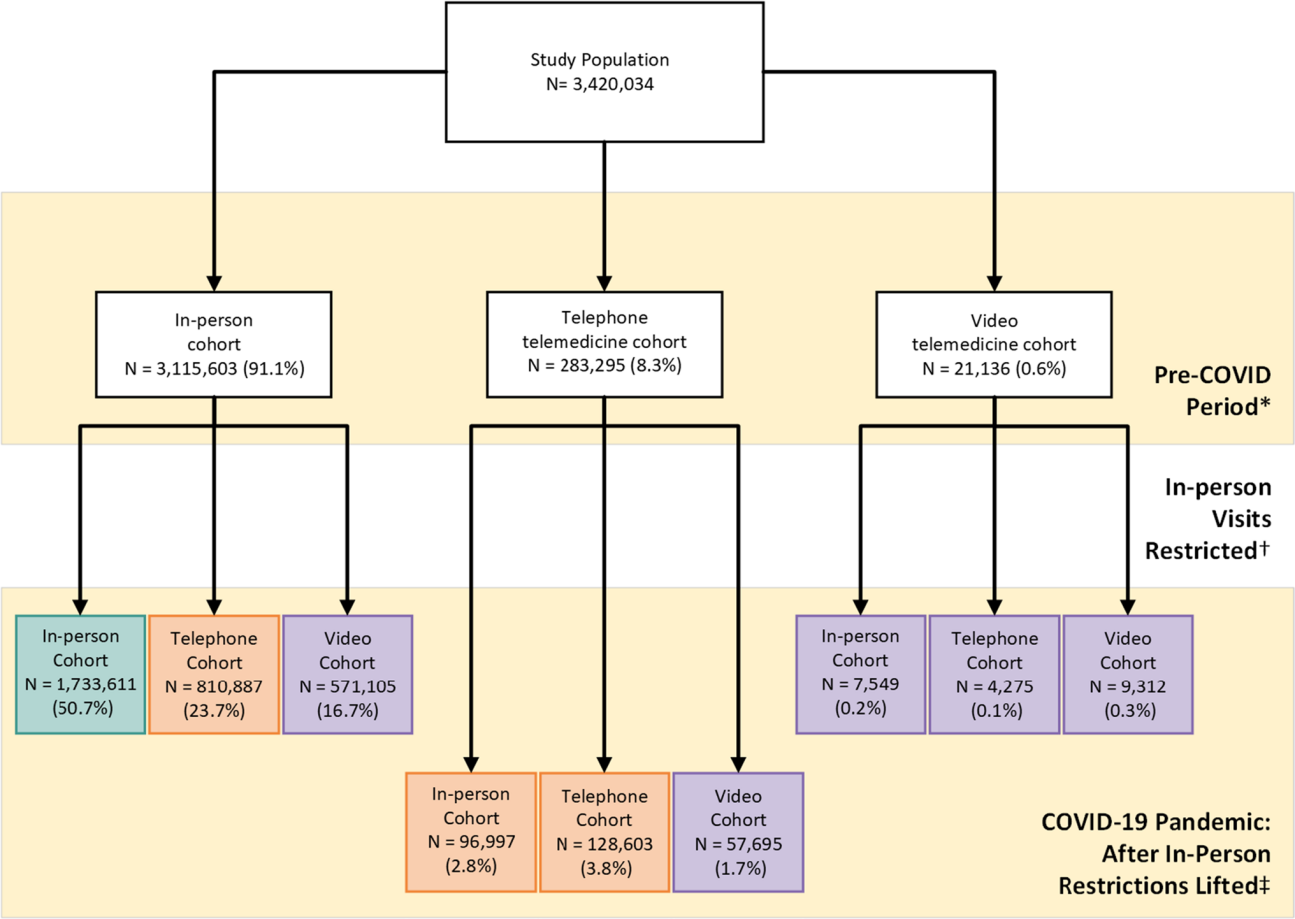
Number of veterans in each primary care visit modality group before the COVID-19 pandemic and after VA Medical Centers re-opened. *The pre-COVID-19 period is March 1, 2019–February 28, 2020. ^†^The period during the COVID-19 pandemic where in-person visits were restricted is March 1, 2020–September 30, 2020. ^‡^The period during the COVID-19 pandemic after in-person visit restrictions were lifted is October 1, 2020–September 30, 2021.

**Figure 2:**
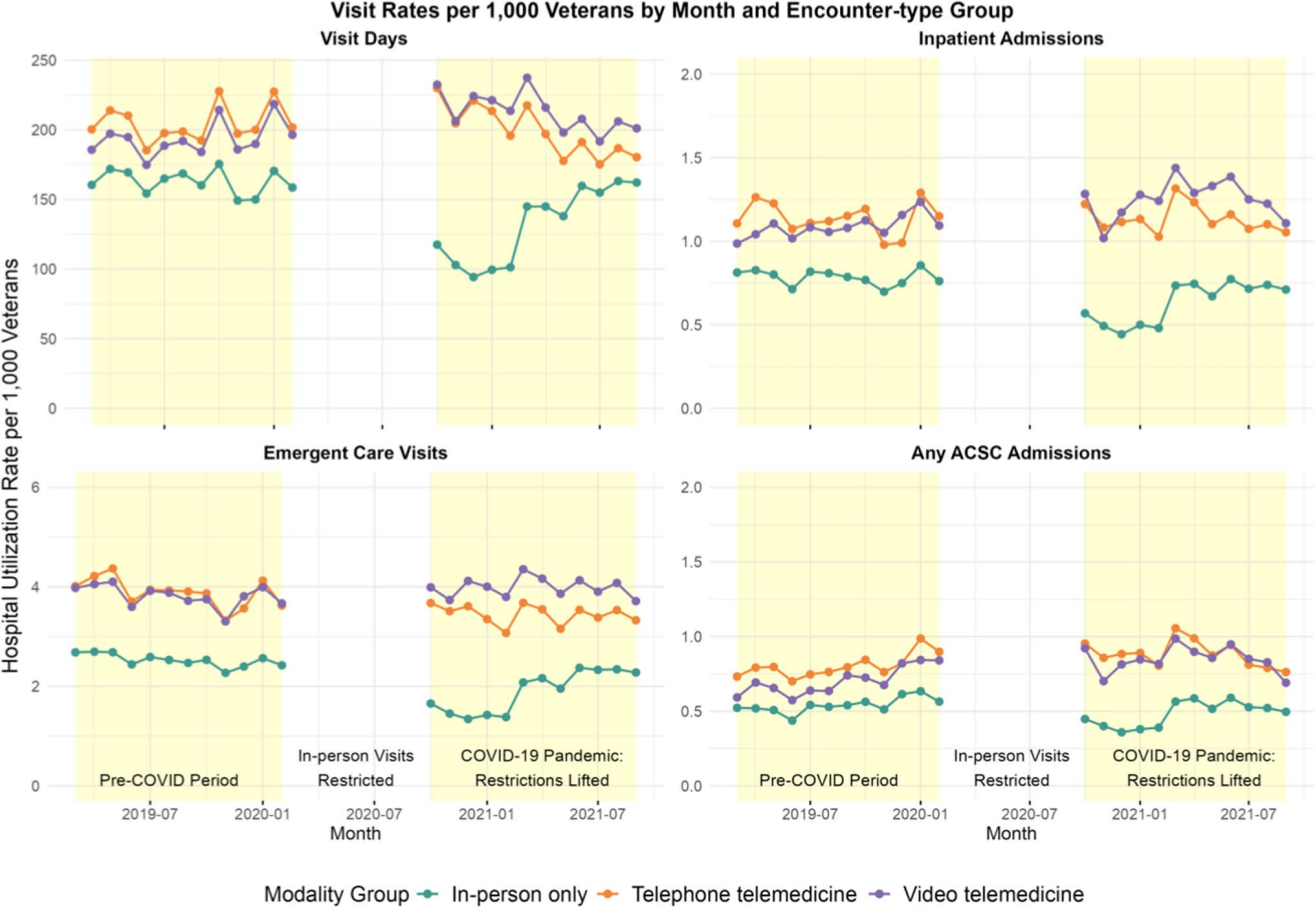
Utilization rate per 1000 veterans by modality group for visit days, emergent care visits, in-patient visits, and ACSC visits within 7 days of index visit.

Prior to the COVID-19 pandemic, 91.1% of veterans visited PC only in-person (*N* = 3,115,603) whereas 283,295 veterans experienced ≥ 1 telephone visit (8.3%), and 21,136 experienced ≥ 1 video visit (0.6%) (Fig. 1). However, after in-person visit restrictions were lifted, 1,381,992 (44.3%) veterans who had only used in-person visits pre-COVID experienced a PC visit by telephone (23.7%) or video (16.7%). Overall healthcare utilization rates over time among video or telephone users were greater than for those using only in-person visits (Fig. 2).

When considering healthcare utilization within 7 days of an index visit by modality group, video versus in-person only users experienced more PC visit days (mean [SD], 4.9 [2.8] vs 3.5 [2.4]; *p* < 0.001) and higher rates per 1000 veterans of emergent care visits (mean [SD]: 15.1 [63.0] vs 11.2 [60.8]; *p* < 0.001), inpatient admissions (mean [SD]: 4.2 [32.3] vs 3.3 [32.1]; *p* < 0.001), and ACSC admissions (mean [SD]: 2.7 [22.9] vs 2.3 [24.2]; *p* < 0.001) (Table 2). Similar rates were reported for the telephone telemedicine versus in-person only group. Similar results were obtained using a 28-day follow-up window from index visit.

**Table 2:** Unadjusted Mean (SD) Estimates for Healthcare Use Stratified by Modality Group and Compared to the In-Person Only Modality Group

	In-person only* N = 1,733,611	Telephone telemedicine *N* = 1,036,487	Video telemedicine *N* = 649,936
7-day follow-up from index visit			
Number of primary care visits days^†^	3.5 (2.4)	4.8 (2.7)	4.9 (2.8)
Number of index primary care visits	3.5 (1.8)	4.6 (2.4)	4.7 (2.5)
Emergent care^‡^ visit rate/1000 veterans	11.2 (60.8)	14.2 (62.2)	15.1 (63.0)
Inpatient admission^§^ rate/1000 veterans	3.3 (32.1)	4.1 (32.1)	4.2 (32.3)
Any ACSC^║^ admission rate/1000 veterans	2.3 (24.2)	3.0 (24.5)	2.7 (22.9)
28-day follow-up from index visit			
Number of primary care visits days	3.5 (2.4)	4.8 (2.7)	4.9 (2.8)
Number of index primary care visits	3.3 (1.5)	4.2 (1.9)	4.2 (1.9)
Emergent care visit rate/1000 veterans	33.8 (128.1)	43.1 (139.8)	46.4 (140.1)
Inpatient admission rate/1000 veterans	9.8 (64.4)	13.0 (70.0)	13.1 (69.5)
Any ACSC^║^ admission rate/1000 veterans	8.3 (50.2)	11.5 (54.6)	10.3 (51.5)

^*^All comparisons versus the in-person only group were significant at the < 0.001 level

^†^The number of intermediate days following an index visit and within the given follow-up window where another primary care visit occurred

^‡^Emergent care includes both emergency department and urgent care visits within the stated follow-up window of an index primary care visit

^§^Inpatient admission within the stated follow-up window of an index primary care visit

^║^*ACSC* = ambulatory care sensitive conditions within the stated follow-up window of an index primary care visit

In adjusted analyses, veterans using video versus exclusively in-person visits pre-pandemic displayed higher odds of emergent care visits (OR = 1.18, 95% CI = 1.16–1.19), inpatient admission (OR = 1.29, 95% CI = 1.25–1.32), and ACSC admissions (OR = 1.30, 95% CI = 1.27–1.34) (Table 3); odds remained statistically significantly higher among telephone versus in-person only groups, though to a lesser degree. Full model output is provided in Appendix 2. Among in-person only users, the COVID-19 pandemic was associated with a decrease in the odds of emergent care (OR = 0.91, 95% CI = 0.90–0.92) and inpatient admission (OR = 0.96, 95% CI = 0.95–0.98), but increased odds of ACSC (OR = 1.04, 95% CI = 1.02–1.06). Finally, the association of being in the video versus in-person only group modified by the COVID-19 pandemic increased the odds of emergent care (OR = 1.08, 95% CI = 1.06–1.10), inpatient admissions (OR = 1.24, 95% CI = 1.20–1.28), and ACSC admission (OR = 1.33, 95% CI = 1.28–1.38); similar but smaller differences were noted among telephone versus in-person only groups. The full interaction model is provided in Appendix 3. Results using a 28-day follow-up window are reported in Table 4 with similar conclusions. Results were also similar when visits with a COVID-19 diagnosis were excluded (Appendix 4).

**Table 3:** Odds Ratio and 95% Confidence Intervals Predicting Healthcare Utilization Outcomes: (1) Emergent Care, (2) Inpatient Admission, and (3) Ambulatory Care Sensitive Condition Within 7 Days of a Primary Care Visit

	Healthcare utilization outcome OR* (95% CI)		
	Emergent care^†^	Inpatient admission^‡^	ACSC^§^ admission
Model 1:			
Modality group			
Telephone telemedicine	1.16 (1.14, 1.17)	1.18 (1.15, 1.20)	1.23 (1.20, 1.25)
Video telemedicine	1.18 (1.16, 1.19)	1.29 (1.25, 1.32)	1.30 (1.27, 1.34)
In-person	Ref	Ref	Ref
COVID-19 pandemic indicator^║^			
During COVID-19 pandemic	0.91 (0.90, 0.92)	0.96 (0.95, 0.98)	1.04 (1.02, 1.06)^#^
Pre-COVID-19 pandemic	Ref	Ref	Ref
Model 2: effect modification with modality group and COVID-19 interaction term			
Telephone telemedicine group during COVID-19 pandemic**	1.04 (1.03, 1.06)	1.12 (1.09, 1.15)	1.26 (1.23, 1.30)
Video telemedicine group during COVID-19 pandemic**	1.08 (1.06, 1.10)	1.24 (1.20, 1.28)	1.33 (1.28, 1.38)
In-person only group during COVID-19 pandemic	0.90 (0.89, 0.91)	0.90 (0.87, 0.93)	0.98 (0.95, 1.01)^¶^
Telephone telemedicine group pre-COVID-19 pandemic	1.16 (1.14, 1.18)	1.13 (1.10, 1.16)	1.17 (1.13, 1.20)
Video telemedicine group pre-COVID-19 pandemic	1.15 (1.13, 1.17)	1.21 (1.17, 1.24)	1.25 (1.21, 1.30)
In-person only group pre-COVID-19 pandemic	Ref	Ref	Ref

^*^OR (odds ratio) is based on a generalized estimating equation model using a logit link function to evaluate differences in hospital utilization outcomes occurring within 7 days of an index primary care visit, adjusted for patient gender, race, ethnicity, rural residence, broadband category, comorbidity score, age, area deprivation index, and social vulnerability index, as well as the number of subsequent primary care visits in the follow-up window delivered in-person, by telephone, or by video

^†^Emergent care includes emergency department and urgent care visits within 7 days of index primary care visit

^‡^Inpatient admission within 7 days of index primary care visit

^§^*ACSC* = ambulatory care sensitive conditions within 7 days of index primary care visit

^║^Before the COVID-19 pandemic (March 1, 2019–February 28, 2020) and after the re-opening of VHA medical centers to in-person visits (October 1, 2020–September 30, 2021)

^¶^Non-significant *p*-value;* p* > 0.05

^#^*p* < 0.01, unless otherwise noted *p* < 0.001

^**^The odds ratios here represent the linear combination of modality group, the pandemic indicator, and the interaction of these two terms for the group reported in comparison to the in-person only group in the pre-pandemic period

**Table 4:** Odds Ratio and 95% Confidence Intervals Predicting Healthcare Utilization Outcomes: (1) Emergent Care, (2) Inpatient Admission, and (3) Ambulatory Care Sensitive Condition Within 28 Days of a Primary Care Visit

	Healthcare utilization outcome OR* (95% CI)		
	Emergent care^†^	Inpatient admission^‡^	ACSC^§^ admission
Model 1:			
Modality group			
* Telephone telemedicine*	1.10 (1.09, 1.11)	1.14 (1.12, 1.16)	1.20 (1.18, 1.21)
* Video telemedicine*	1.14 (1.13, 1.15)	1.23 (1.21, 1.25)	1.26 (1.24, 1.28)
* In-person*	Ref	Ref	Ref
COVID-19 pandemic indicator^║^			
* During COVID-19 pandemic*	0.93 (0.92, 0.94)	1.00 (0.99, 1.02)^¶^	1.22 (1.20, 1.23)
* Pre-COVID-19 pandemic*	Ref	Ref	Ref
Model 2: effect modification with modality group and COVID-19 interaction term			
Telephone telemedicine group during COVID-19 pandemic**	1.02 (1.01, 1.03)	1.14 (1.12, 1.16)	1.45 (1.43, 1.48)
Video telemedicine group during COVID-19 pandemic**	1.07 (1.05, 1.08)	1.24 (1.21, 1.27)	1.51 (1.48, 1.54)
* In-person only group during COVID-19 pandemic*	0.91 (0.91, 0.92)	0.97 (0.96, 0.99)	1.17 (1.15, 1.19)
* Telephone telemedicine group pre-COVID-19 pandemic*	1.09 (1.08, 1.11)	1.11 (1.10, 1.14)	1.15 (1.13, 1.17)
* Video telemedicine group pre-COVID-19 pandemic*	1.12 (1.11, 1.13)	1.19 (1.17, 1.22)	1.23 (1.21, 1.26)
* In-person only group pre-COVID-19 pandemic*	Ref	Ref	Ref

^*^OR (odds ratio) is based on a generalized estimating equation model using a logit link function to evaluate differences in hospital utilization outcomes occurring within 28 days of an index primary care visit, adjusted for patient gender, race, ethnicity, rural residence, broadband category, comorbidity score, age, area deprivation index, and social vulnerability index, as well as the number of subsequent primary care visits in the follow-up window delivered in-person, by telephone, or by video

^†^Emergent care includes emergency department and urgent care visits within 28 days of index primary care visit

^‡^Inpatient admission within 28 days of index primary care visit

^§^*ACSC* = ambulatory care sensitive conditions within 28 days of index primary care visit

^║^Before the COVID-19 pandemic (March 1, 2019–February 28, 2020) and after the re-opening of VHA medical centers to in-person visits (October 1, 2020–September 30, 2021)

^¶^Non-significant *p*-value; *p* > 0.05

^#^*p* < 0.01, unless otherwise noted *p* < 0.001

The odds ratios here represent the linear combination of modality group, the pandemic indicator, and the interaction of these two terms for the group reported in comparison to the in-person only group in the pre-pandemic period

## DISCUSSION

The COVID-19 pandemic altered how VHA healthcare was delivered nationwide. However, as restrictions receded, telemedicine has remained an important delivery modality. In this study, we examined whether follow-up healthcare utilization differed between veterans receiving PC exclusively in-person or with at least one telephone or video visit. In this study we found (1) the number of veterans receiving PC in a combination of modalities has substantially increased, and (2) those using at least some video telemedicine tend to be younger, female, and urban-residing, but (3) have higher adjusted odds of experiencing emergent visits, inpatient admissions, or ACSC admissions within 7 days of an index PC visit. As telemedicine use, both via telephone and video, continues to be a routine part of care, our findings highlight the importance of understanding not only who is using telemedicine, but how such use works in conjunction with in-person visits and the subsequent effect on downstream care.

While telemedicine was available before the pandemic,^27–29^ it rapidly expanded at the onset of the COVID-19 pandemic.^30–32^ Overall, the ability to access care in multiple ways has the potential to improve access, especially among women and younger populations^3, 4^ who may feel uncomfortable receiving care in-person, require childcare, or lack the ability to take time off work. For many, especially those living in rural or underserved areas, the time and travel savings are a significant benefit despite growing literature about inequitable access to broadband in rural locations.^33^ However, if telemedicine use is related to further downstream utilization, it might not save the patient time or money and could cost healthcare systems more.

We found video telemedicine users had significantly more hospital utilization following a PC visit. This is contrary to previous studies indicating more readily available PC or urgent care access (in-person or telemedicine) could potentially reduce use of emergency healthcare resources.^34–36^ However, patients participating in the study by Huang et al. could self-select between video and telephone visits when in-person was only available after either modality was first used. In this setting, non-tech savvy patients could choose phone to facilitate an in-person visit, which could improve the outcomes related to a video visit. Further, with the increase in subsequent healthcare utilization noted in our study, it is possible a system already exacerbated by the COVID-19 pandemic may see additional system-wide strains in the forms of staff, supply, and space shortages.^37^ This strain was especially apparent at pandemic start; however, ongoing shortages of nursing staff, medications, and other supplies^38–40^ continue to impact healthcare.

It is not yet clear why patients who utilized telemedicine had significantly higher odds of emergent or inpatient care, and ACSC admissions even after adjusting for patient characteristics, including comorbidity levels. Because we categorized patients based on their overall telemedicine use across both study periods, it is important to note this increased use does not imply care received over the phone or via video was inappropriate. Instead, we hypothesize telemedicine users may choose to use this modality multiple reasons. For example, both telemedicine groups had a higher number of intermediate PC visit days, representing increased PC use in between index visits, and yet showed similar average comorbidity scores. It is likely some video telemedicine patients requiring emergent or inpatient care may generally use the healthcare system more and thus take advantage of multiple modalities, while others may use this modality because they are healthy and do not feel an in-person visit is needed or find a video visit more convenient. It is also possible that patients initiated or agreed to try telemedicine for the first time because of an emerging acute need. This may have been especially true during so-called COVID waves, when patients were more likely to seek telemedicine visits to avoid the risk of in-person appointments. Subsequent PC visits would be expected to be higher if telemedicine cannot resolve the issue or if it determined a physical exam or procedure, or other follow-up is required. In this context, telemedicine may act as an intermediate facilitator or triaging agent for important and life-saving care that may otherwise have been delayed or overlooked. This may be especially true for patients who want to receive care within the VHA versus obtaining VHA-funded care in their community. Future work should consider the factors that lead a patient to choose telemedicine, how telemedicine and in-person visits work in conjunction with one another, and the effect of facility-level factors influencing patient flow from PC visit to emergent care or inpatient admission and telemedicine utilization patterns.

This research has limitations. First, our results amongst the veteran population may not be generalizable to the overall US adult population. However, the size of this nationwide study remains a significant strength. Second, we were unable to account for any healthcare utilization obtained in community locations. This may be particularly relevant for veterans who have private insurance or may live too far away from VHA services to quickly obtain emergency services at a VHA medical center. Selection bias is also a concern as telemedicine users may be more connected to their VHA healthcare team and thus more likely to seek care within the VHA system. Finally, we did not determine if the use of emergent care or inpatient services was related to the chief complaint of the PC index visit. However, it remains likely that follow-up healthcare in emergent or inpatient settings is related to acute exacerbations of chronic conditions addressed at nearly every PC visit including diabetes, hypertension, and heart failure. Reviewing both index visits and follow-up healthcare complaints could provide insight into whether appropriate care was missed during the index visit leading to further utilization later.

The COVID-19 pandemic considerably increased telemedicine use. Identifying and studying veterans with a measurable difference in healthcare utilization can provide insights into how the method of care delivery may impact PC access and follow-up healthcare utilization.

### Supplementary Information

Below is the link to the electronic supplementary material.

Supplementary file1 (DOCX 66 kb)

## Data Availability

The datasets generated and analyzed are not publicly available due to VHA privacy and confidentiality requirements.

## Declarations:

### Disclaimer:

The funders had no role in the design and conduct of the study; collection, management, analysis, and interpretation of the data; preparation, review, or approval of the manuscript; and decision to submit the manuscript for publication.
