# Supplementary material for: Healthcare Utilization Differences Among Primary Care Patients Using Telemedicine in the Veterans Health Administration: a Retrospective Cohort Study
Source: J Gen Intern Med. 2024 Jan 22;39(Suppl 1):109–17. doi: 10.1007/s11606-023-08472-1 (PMC10937860; doi:10.1007/s11606-023-08472-1)
Supplement: Supplementary file 1 — Supplementary file1 (DOCX 66 kb) [file 11606_2023_8472_MOESM1_ESM.docx]

**Appendix A. Primary Care Stop Code Classifications**

Primary care encounters were categorized using stop codes, a pair of proprietary three-digit codes assigned to each outpatient encounter. Within the corporate data warehouse, outpatient visits are designated with both primary and secondary stop codes. Both are needed to identify applicable primary care visits and determine the modality of these visits.

To start, the following primary stop codes are included.

These primary stop codes in combination with the listed secondary stop codes are excluded.

**Table A.1 Primary and Secondary stop codes used to identify a primary care visit.**

| **Primary Stop Code** | **Primary Stop Code Description** | **Secondary Stop Codes to Exclude** | **Secondary Stop Code Description** |
| --- | --- | --- | --- |
| 322 | COMP WOMEN'S HLTH | 107 | EKG |
| 323 | PRIMARY CARE/MEDICINE | 115 | ULTRASOUND |
| 338 | TELEPHONE  PRIMARY CARE | 152 | ANGIOGRAM CATHETERIZATION |
| 349 | SLEEP MEDICINE | 311 | PACEMAKER |
| 350 | GERIPACT | 321 | GI ENDOSCOPY |
| 704 | WMS SPECIFIC  PREVENTIVE CARE | 328 | MEDICAL/SURGICAL  DAY UNIT |
|  |  | 329 | MEDICAL PROCEDURE UNIT |
|  |  | 333 | CARDIAC CATHETERIZATION |
|  |  | 334 | CARDIAC STRESS TEST |
|  |  | 430 | CYSTO ROOM IN UROLOGY |
|  |  | 435 | SURGICAL PROCEDURE |
|  |  | 474 | RESEARCH |
|  |  | 710 | PREVENTIVE IMMUNIZATION |
|  |  | 719 | MHV SECURE MESSAGING |
|  |  | 999 | OCCUPATIONAL HEALTH |

Once these primary care visits are identified, we categorized them by how they were delivered (i.e., in-person, via telephone, or by video). This is through a combination of primary and secondary stop codes. Notably, we have included clinical video telehealth visits as an in-person modality, as veterans come into the VA clinic but connect with a VA provider in a different location. They are very similar to a face-to-face visit in the process of care.

**Table A.2 Primary and Secondary stop codes used to identify the modality of a primary care visit**

| **Visit Modality** | **Primary Stop Code** | **Primary Stop Code Description** | **Secondary Stop Code** | **Secondary Stop Code Description** |
| --- | --- | --- | --- | --- |
| Telephone | 338 | TELEPHONE PRIMARY CARE | 185 | NURSE PRACTITIONER |
|  |  |  | 186 | PHYSICIAN ASSISTANT |
|  |  |  | 187 | CLINICAL NURSE SPECIALIST |
|  |  |  | 188 | FELLOW/RESIDENT |
| Video | 322 | COMP WOMEN'S HLTH | 179 | RT CLIN VID CARE HOME |
|  | 323 | PRIMARY CARE/MEDICINE |  |  |
|  | 349 | SLEEP MEDICINE |  |  |
|  | 350 | GERIPACT |  |  |
|  | 704 | WMS SPECIFIC  PREVENTIVE CARE |  |  |
| In-Person |  | | 185 | NURSE PRACTITIONER |
|  |  |  | 186 | PHYSICIAN ASSISTANT |
|  |  |  | 187 | CLINICAL NURSE SPECIALIST |
|  |  |  | 188 | FELLOW/RESIDENT |
|  | 322 | COMP WOMEN'S HLTH | 690 | RT CLIN VID TH PAT SITE |
|  | 323 | PRIMARY CARE/MEDICINE | 692 | CVT PRV SITE SAME DIV/STA |
|  | 349 | SLEEP MEDICINE | 693 | RT CLIN VD TH PRV SITE (DIFSTA) |
|  | 350 | GERIPACT | 694 | SF TH PAT SITE |
|  | 704 | WMS SPECIFIC  PREVENTIVE CARE | 695 | SF TH PRV SITE SAME DIV/STA |
|  |  | | 696 | SF TH PRV SITE (DIFSTA) |
|  |  |  | 697 | CHART CONSULT |
|  |  |  | 698 | REMOTE PT MONITOR PROV SITE |
|  |  |  | 699 | CVT EMERGENCY CONSULT |

**Appendix B. Complete Model Output for Model #1 from Table 3 without the Modality Group and COVID-19 Indicator Interaction Term and using a 7-day follow-up window from an Index Visit**

|  | **Healthcare Utilization Outcome OR* (95% CI)** | | |
| --- | --- | --- | --- |
|  | **Emergent Care**^†^ | **Inpatient Admission**^‡^ | **ACSC**^§^ **Admission** |
| **Modality Group**  *(Reference: In-Person)* |  |  |  |
| *Telephone Telemedicine* | 1.16 (1.14, 1.17) | 1.18 (1.16, 1.21) | 1.23 (1.20, 1.26) |
| *Video Telemedicine* | 1.18 (1.16, 1.19) | 1.29 (1.25, 1.32) | 1.31 (1.27, 1.34) |
| **COVID-19 Pandemic Indicator**^║^  *(Reference: Pre-COVID)* | 0.91 (0.90, 0.92) | 0.96 (0.95, 0.98) | 1.03 (1.01, 1.05) |
| **Visits Within 7-days of Index Visits,** *1-Visit Increase* |  |  |  |
| *Occurring In-person* | 2.26 (2.08, 2.45) | 2.70 (2.44, 2.98) | 2.80 (2.54, 3.08) |
| *Occurring by Telephone Telemedicine* | 2.29 (2.24, 2.35) | 2.04 (1.98, 2.11) | 2.23 (2.16, 2.31) |
| *Occurring by Video Telemedicine* | 1.59 (1.51, 1.67) | 1.11 (0.99, 1.26) | 1.42 (1.26, 1.61) |
| **Gender** *(Reference: Male)* |  |  |  |
| *Female* | 1.09 (1.07, 1.11) | 0.89 (0.86, 0.92) | 0.79 (0.75, 0.83) |
| **Race** *(Reference: White)* |  |  |  |
| *American Indian* | 1.13 (1.08, 1.19) | 1.07 (0.98, 1.18) | 1.12 (1.02, 1.24) |
| *Asian* | 0.74 (0.70, 0.78) | 0.62 (0.55, 0.69) | 0.67 (0.59, 0.77) |
| *Black/ African American* | 1.29 (1.27, 1.30) | 1.10 (1.07, 1.13) | 1.05 (1.02, 1.08) |
| *Native Hawaiian/ Pacific Islander* | 0.90 (0.85, 0.95) | 0.82 (0.75, 0.90) | 0.95 (0.86, 1.05) |
| *Missing* | 0.93 (0.91, 0.96) | 0.87 (0.83, 0.92) | 0.86 (0.82, 0.90) |
| **Ethnicity**  *(Reference: Not Hispanic)* |  |  |  |
| *Hispanic/Latino* | 1.03 (1.00, 1.06) | 0.85 (0.80, 0.91) | 0.87 (0.80, 0.93) |
| *Missing* | 0.89 (0.86, 0.91) | 0.88 (0.83, 0.93) | 0.90 (0.85, 0.96) |
| **Rurality**^¶^ *(Reference: Urban)* |  |  |  |
| *Rural* | 0.67 (0.66, 0.68) | 0.71 (0.69, 0.72) | 0.81 (0.79, 0.82) |
| **Broadband Category**  *(Reference: Optimal)* |  |  |  |
| *Inadequate* | 1.01 (0.99, 1.03) | 0.99 (0.95, 1.03) | 1.05 (1.01, 1.09) |
| *Adequate* | 0.95 (0.94, 0.96) | 0.96 (0.94, 0.98) | 1.01 (0.99, 1.03) |
| **QUAN Comorbidity Score,**  **1 unit increase** | 1.23 (1.23, 1.24) | 1.32 (1.31, 1.33) | 1.31 (1.30, 1.32) |
| **Age**, 10-unit increase | 0.96 (0.96, 0.97) | 1.12 (1.11, 1.13) | 1.25 (1.24, 1.26) |
| **ADI**^#^, 10-unit increase | 1.00 (1.00, 1.00) | 1.03 (1.02, 1.03) | 1.06 (1.06, 1.07) |
| **SVI**^**^, 1-unit increase | 1.37 (1.35, 1.40) | 1.28 (1.23, 1.32) | 1.00 (1.00, 1.00) |

* OR (odds ratio) is based on a Generalized Estimating Equation model using a logit link function to evaluate differences in hospital utilization outcomes occurring within 7-days of an index primary care visit.

† Emergent Care includes both emergency department and urgent care visits within 7 days of index primary care visit

‡ Inpatient Admission within 7-days of index primary care visit.

§ ACSC = Ambulatory Care Sensitive Conditions within 7-days of index primary care visit.

║Before the COVID-19 Pandemic (March 1, 2019-February 28, 2020) and after the re-opening of VHA medical centers to in-person visits (October 1, 2020-September 30, 2021).

¶Includes patients who lived in a rural area at any time during the study period.

#ADI=Area Deprivation Index, ranks neighborhoods by socioeconomic disadvantage on a scale of 0-100 with lower rankings indicating less social disadvantage

** Social Vulnerability Index, the overall census tract ranking determined by the U.S. Centers for Disease Control and Prevention to identify communities at higher social risk following a disaster

**Appendix C. Complete Model Output for Model #2 reported in Table 3 inclusive the Modality Group and COVID-19 Indicator Interaction Term and using a 7-day follow-up window from an Index Visit**

|  | **Healthcare Utilization Outcome OR* (95% CI)** | | |
| --- | --- | --- | --- |
|  | **Emergent Care**^†^ | **Inpatient Admission**^‡^ | **ACSC**^§^ **Admission** |
| **Effect Modification with Modality Group & COVID-19 Interaction Term** |  |  |  |
| *Telephone Telemedicine Group During COVID-19 Pandemic*^║^ | 1.04 (1.03, 1.06) | 1.12 (1.09, 1.15) | 1.26 (1.23, 1.30) |
| *Video Telemedicine Group During COVID-19 Pandemic*^║^ | 1.08 (1.06, 1.10) | 1.24 (1.20, 1.28) | 1.33 (1.28, 1.38) |
| *In-Person Only Group*  *During COVID-19*  *Pandemic* | 0.90 (0.89, 0.91) | 0.90 (0.87, 0.93) | 0.98 (0.95, 1.01)^††^ |
| *Telephone Telemedicine*  *Group Pre-COVID-19*  *Pandemic* | 1.16 (1.14, 1.18) | 1.13 (1.10, 1.16) | 1.17 (1.13, 1.20) |
| *Video Telemedicine*  *Group Pre-COVID-19*  *Pandemic* | 1.15 (1.13, 1.17) | 1.21 (1.17, 1.24) | 1.25 (1.21, 1.30) |
|  | Ref | Ref | Ref |
| **Visits in Between Index Visits,** *1-Visit Increase* |  |  |  |
| *Occurring In-person* | 2.26 (2.08, 2.45) | 2.74 (2.49, 3.03) | 2.84 (2.59, 3.12) |
| *Occurring by Telephone Telemedicine* | 2.30 (2.25, 2.35) | 2.04 (1.98, 2.11) | 2.23 (2.16, 2.31) |
| *Occurring by Video Telemedicine* | 1.57 (1.49, 1.65) | 1.14 (1.02, 1.27) | 1.45 (1.29, 1.62) |
| **Gender** *(Reference: Male)* |  |  |  |
| *Female* | 1.09 (1.07, 1.11) | 0.89 (0.86, 0.92) | 0.78 (0.75, 0.82) |
| **Race** *(Reference: White)* |  |  |  |
| *American Indian* | 1.13 (1.08, 1.19) | 1.07 (0.98, 1.18) | 1.12 (1.02, 1.24) |
| *Asian* | 0.74 (0.70, 0.78) | 0.62 (0.55, 0.69) | 0.67 (0.59, 0.77) |
| *Black/ African American* | 1.29 (1.27, 1.30) | 1.10 (1.07, 1.13) | 1.05 (1.02, 1.08) |
| *Native Hawaiian/ Pacific Islander* | 0.90 (0.85, 0.95) | 0.82 (0.75, 0.90) | 0.95 (0.86, 1.05) |
| *Missing* | 0.93 (0.91, 0.96) | 0.87 (0.83, 0.92) | 0.86 (0.82, 0.90) |
| **Ethnicity**  *(Reference: Not Hispanic)* |  |  |  |
| *Hispanic/Latino* | 1.03 (1.00, 1.06) | 0.85 (0.80, 0.91) | 0.87 (0.80, 0.93) |
| *Missing* | 0.89 (0.86, 0.91) | 0.88 (0.83, 0.93) | 0.90 (0.85, 0.96) |
| **Rurality**^¶^ *(Reference: Urban)* |  |  |  |
| *Rural* | 0.67 (0.66, 0.68) | 0.71 (0.69, 0.72) | 0.81 (0.79, 0.82) |
| **Broadband Category**  *(Reference: Optimal)* |  |  |  |
| *Inadequate* | 1.01 (0.99, 1.03) | 0.99 (0.95, 1.03) | 1.05 (1.01, 1.09) |
| *Adequate* | 0.95 (0.94, 0.96) | 0.96 (0.94, 0.98) | 1.01 (0.99, 1.03) |
| **QUAN Comorbidity Score,**  **1 unit increase** | 1.23 (1.23, 1.23) | 1.32 (1.31, 1.33) | 1.31 (1.30, 1.32) |
| **Age**, 10-unit increase | 0.96 (0.96, 0.97) | 1.12 (1.11, 1.13) | 1.25 (1.24, 1.26) |
| **ADI**^#^, 10-unit increase | 1.00 (1.00, 1.00) | 1.03 (1.02, 1.03) | 1.06 (1.06, 1.07) |
| **SVI****, 1-unit increase | 1.37 (1.35, 1.40) | 1.28 (1.23, 1.32) | 1.00 (1.00, 1.00) |

* OR (odds ratio) is based on a Generalized Estimating Equation model using a logit link function to evaluate differences in hospital utilization outcomes occurring within 7-days of an index primary care visit.

† Emergent Care includes both emergency department and urgent care visits within 7 days of index primary care visit

‡ Inpatient Admission within 7 days of index primary care visit.

§ ACSC = Ambulatory Care Sensitive Conditions within 7 days of index primary care visit.

║ The odds ratios here represent the linear combination of modality group, the pandemic indicator, and the interaction of these two terms for the group reported in comparison to the in-person only group in the pre-pandemic period.

¶ Includes patients who lived in a rural area at any time during the study period.

# ADI=Area Deprivation Index, ranks neighborhoods by socioeconomic disadvantage on a scale of 0-100 with lower rankings indicating less social disadvantage

** Social Vulnerability Index, the overall census tract ranking determined by the U.S. Centers for Disease Control and Prevention to identify communities at higher social risk following a disaster

†† Non-significant p-value; p>0.05, unless otherwise noted p<0.001

**Appendix D. Odds Ratio and 95% Confidence Intervals Predicting Healthcare Utilization Outcomes: 1) Emergent Care, 2) Inpatient Admission, and 3) Ambulatory Care Sensitive Condition within 7-Days of a Primary Care Visit, Excluding Visits with a COVID-19 Diagnosis.**

|  | **Healthcare Utilization Outcome OR* (95% CI)** | | |
| --- | --- | --- | --- |
|  | **Emergent Care**^†^ | **Inpatient Admission**^‡^ | **ACSC**^§^ **Admission** |
| **Model 1:** |  |  |  |
| **Modality Group** |  |  |  |
| *Telephone Telemedicine* | 1.15 (1.14, 1.16) | 1.17 (1.14, 1.19) | 1.22 (1.19, 1.25) |
| *Video Telemedicine* | 1.16 (1.15, 1.18) | 1.27 (1.24, 1.30) | 1.30 (1.26, 1.33) |
| *In-Person* | Ref | Ref | Ref |
| **COVID-19 Pandemic Indicator**^║^ |  |  |  |
| *During COVID-19 Pandemic* | 0.87 (0.87, 0.88) | 0.91 (0.90, 0.93) | 1.00 (0.98, 1.02) ^¶^ |
| *Pre-COVID-19 Pandemic* | Ref | Ref | Ref |
| **Model 2: Effect Modification with Modality Group & COVID-19 Interaction Term** |  |  |  |
| *Telephone Telemedicine Group During COVID-19 Pandemic*** | 0.99 (0.98, 1.01) | 1.05 (1.02, 1.08) | 1.21 (1.18, 1.25) |
| *Video Telemedicine Group During COVID-19 Pandemic*** | 1.03 (1.01, 1.05) | 1.16 (1.13, 1.20) | 1.27 (1.23, 1.32) |
| *In-Person Only Group During COVID-19 Pandemic* | 0.88 (0.87, 0.89) | 0.87 (0.84, 0.89) | 0.95 (0.92, 0.98) |
| *Telephone Telemedicine Group Pre-COVID-19 Pandemic* | 1.17 (1.15, 1.18) | 1.13 (1.10, 1.16) | 1.17 (1.13, 1.20) |
| *Video Telemedicine Group Pre-COVID-19 Pandemic* | 1.15 (1.13, 1.17) | 1.21 (1.17, 1.25) | 1.26 (1.21, 1.30) |
| *In-Person Only Group*  *Pre-COVID-19 Pandemic* | Ref | Ref | Ref |

* OR (odds ratio) is based on a Generalized Estimating Equation model using a logit link function to evaluate differences in hospital utilization outcomes occurring within 28-days of an index primary care visit, adjusted for patient gender, race, ethnicity, rural residence, broadband category, comorbidity score, age, area deprivation index, and social vulnerability index, as well as the number of subsequent primary care visits in the follow-up window delivered in-person, by telephone, or by video.

† Emergent Care includes emergency department and urgent care visits within 7-days of index primary care visit

‡ Inpatient Admission within 7-days of index primary care visit.

§ ACSC = Ambulatory Care Sensitive Condition within 7-days of index primary care visit.

║Before the COVID-19 Pandemic (March 1, 2019-February 28, 2020) and after the re-opening of VHA medical centers to in-person visits (October 1, 2020-September 30, 2021).

¶ Non-significant p-value; p>0.05, # p<0.01, unless otherwise noted p<0.001

# The odds ratios here represent the linear combination of modality group, the pandemic indicator, and the interaction of these two terms for the group reported in comparison to the in-person only group in the pre-pandemic period.
